# Significance of Cuscutain, a cysteine protease from *Cuscuta reflexa*, in host-parasite interactions

**DOI:** 10.1186/1471-2229-10-227

**Published:** 2010-10-22

**Authors:** Marc Bleischwitz, Markus Albert, Hans-Lothar Fuchsbauer, Ralf Kaldenhoff

**Affiliations:** 1 Pathology of Forest Trees, TU Munich, Am Hochanger 13, 85354 Freising, Germany; 2 ZMBP, Forschungsgruppe Pflanzenbiochemie, Eberhard-Karls-Universität Tübingen, Auf der Morgenstelle 5, D-72076 Tübingen, Germany; 3 University of Applied Sciences, Chemistry and Biotechnology, Schnittspahnstr. 12, 64287 Darmstadt, Germany; 4 Darmstadt University of Technology, Applied Plant Science, Schnittspahnstr. 10, 64287 Darmstadt, Germany

## Abstract

**Background:**

Plant infestation with parasitic weeds like *Cuscuta reflexa *induces morphological as well as biochemical changes in the host and the parasite. These modifications could be caused by a change in protein or gene activity. Using a comparative macroarray approach *Cuscuta *genes specifically upregulated at the host attachment site were identified.

**Results:**

One of the infestation specific *Cuscuta *genes encodes a cysteine protease. The protein and its intrinsic inhibitory peptide were heterologously expressed, purified and biochemically characterized. The haustoria specific enzyme was named cuscutain in accordance with similar proteins from other plants, e.g. papaya. The role of cuscutain and its inhibitor during the host parasite interaction was studied by external application of an inhibitor suspension, which induced a significant reduction of successful infection events.

**Conclusions:**

The study provides new information about molecular events during the parasitic plant - host interaction. Inhibition of cuscutain cysteine proteinase could provide means for antagonizing parasitic plants.

## Background

Parasitic weeds such as *Cuscuta reflexa *are obligate holoparasites with low host specificity. The plants are found in areas with relatively mild climates around the world. In farming regions, these parasites cause substantial damage to many commercially important crops such as sugar beet, alfalfa, pepper, cucumber, tomato potato or allium [1]. Currently, an effective control of *Cuscuta *outbreaks is based on preventive strategies including control of seed contamination and application of herbicides prior to seed emergence. The use of herbicides on infected plants with an established host parasite interaction only appears to be successful and not harmful to the host plant if the host is herbicide resistant [2,3]. Due to difficulties with conventional breeding techniques, molecular biology genomic research on parasites is needed to develop new control strategies [4-8]. Research on host reactions to parasitic plant infection in model plants such as *Arabidopsis thaliana*, *Medicago truncatula *and crops like tomato or tobacco have already generated promising results [9-12].

In *Cuscuta *spp. photosynthesis is reduced or absent [13]. Consequently, the plant depends on carbohydrates withdrawn from the host plant [14]. A connection (haustorium) at the contact site is established through the secretion of enzymes and sticky substances consisting mainly of de-esterified pectins [15]. At early stages of *Cuscuta *invasion, host plants react with specific gene expression to regulate processes including calcium release, cell elongation and cell wall modification (Albert, Werner, Proksch, Fry, & Kaldenhoff 2004; Werner, Uehlein, Proksch, & Kaldenhoff 2001; [16]. A gene coding for an arabinogalactan protein (AGP) was found to be up-regulated in tomato at an early stage of infection and it has a significant function for *C. reflexa *attachment to the host plant (Albert, Belastegui-Macadam, & Kaldenhoff 2006). After attachment, the host is invaded by hyphae and chimeric cell walls of host and *Cuscuta *cells are formed [17]. Phloem and xylem connections transfer water, nitrogen-compounds, assimilates and even RNA, proteins or plant viruses from the host to the parasitic plant [18-20].

The current knowledge about gene expression in the parasite *Cuscuta *at early stages of infection is limited. Besides host responses, parasitic plant reactions need to be determined for a complete elucidation of the infection process. This knowledge will likely be one of the prerequisites for the improvement of strategies to prevent or control *Cuscuta*-infection. For a first overview of parasite responses, we have constructed a *Cuscuta *cDNA-library corresponding to mRNAs specific for early stages of haustoria development. Here, we describe one of the identified genes, which encodes a *Cuscuta reflexa *haustoria specific cysteine protease that we named cuscutain. Its expression, biochemical characteristics and significance during the infection process opens the possibility to develop a cuscutain-based strategy against *Cuscuta *infection.

## Results

### Cuscutain

**Figure 1:**
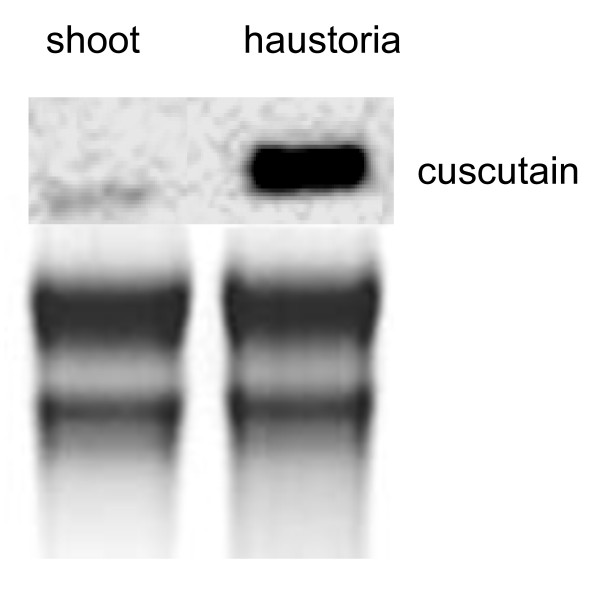
**Northern blot with total *Cuscuta *RNA**. *Cuscuta *RNA from shoots (shoot) or shoot material with haustoria (haustoria). Upper panel: hybridization signal with a cuscutain-cDNA probe. Lower panel: Ethidium bromide stained total RNA to indicate even loading.

**Figure 2:**
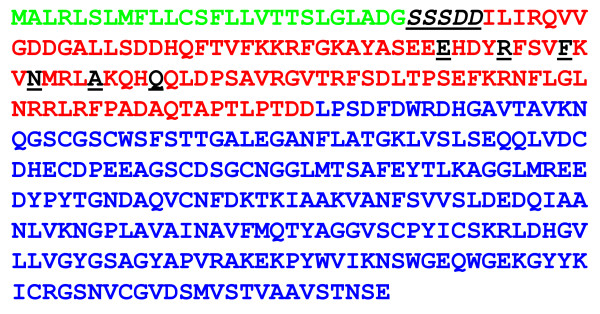
**Predicted cuscutain sequence**. Prepeptide (green), propeptide (red) and the catalytic protein (blue) are depicted as well as the cleavage site and the ERFIN-motif (black, underlined).

mRNA from *Cuscuta *tissue containing haustoria was employed to construct a cDNA library with 7000 primary transformants. Putative haustorium specific expression of the corresponding genes was identified by differential cDNA hybridization (haustorium containing material versus shoot without haustoria) to all obtained cDNA clones assigned on a macroarray. 16 different clones were preselected by this procedure showing a more intense signal on a macroarray chip when hybridized with a cDNA probe from RNA of haustoria containing tissue. One of the signals with a remarkable differential intensity corresponded to a cDNA clone derived from a cysteine proteinase encoding mRNA (Accession No.: FB701665). Because cysteine proteinases were known to participate in some interspecies interactions, we were interested to study the role of this *Cuscuta reflexa *enzyme. For verification of spatial expression, *Cuscuta*-RNA from the two tissue types was further characterized by Northern blot with a probe derived from the above said cDNA clone. Just a faint signal was obtained in RNA from *Cuscuta *shoots lacking haustorial structures and a strong one was obtained in RNA from shoot material with haustoria, which was harvested at three days post attachment (Figure 1). Sequence analysis of the deduced mRNA revealed that translational start and stop codons including poly A tail were cloned as cDNA indicating a full length cDNA-insertion. Sequence comparison obtained from BLAST-n at NCBI revealed a high sequence identity to cysteine proteinases like *Ipomoea batatas *papain-like cysteine proteinase isoform II (86%), *Phaseolus vulgaris *Moldavian encoding cysteine proteinase (78%) or *Arachis hypogaea *cysteine protease-like protein (77%). An equally high identity to numerous cysteine proteinases was observed when the translated sequence was subjected to a comparison with entries from Expasy Proteomics Server at Swiss Institute of Bioinformatics (e.g. 86% overall identity to *Ipomoea batatas *papain-like cysteine proteinase isoform II). According to sequence alignment and protein domain identification tools, specific functional sites could be identified (Figure 2). Hence, the predicted protein consists of a prepeptide, thought to be responsible for its extracellular localization (98.2% probability). The corresponding cleavage site between prepeptide and the subsequent propeptide is represented by a SSSDD sequence (Figure 2, 99.9% probability). Next to the prepeptide from N- to C-terminus the propeptide harbors a so called ERFIN-motif [21], which is a stabilizing component of intramolecular interaction. This protein region was described to form an inhibitor of the proteinase activity [22]. Furthermore the propeptide acts as an intramolecular chaperone [23]. The actual cysteine proteinase region contains characteristic active sites as indicated by motiv search (Prosite, Swiss Institute of Bioinformatics) and comprises a protein with a calculated molecular weight of 24.8 kDa. That of the prepeptide is 11.7 kDa.

### Biochemical characterization of cuscutain and the inhibitory propeptide

**Figure 3:**
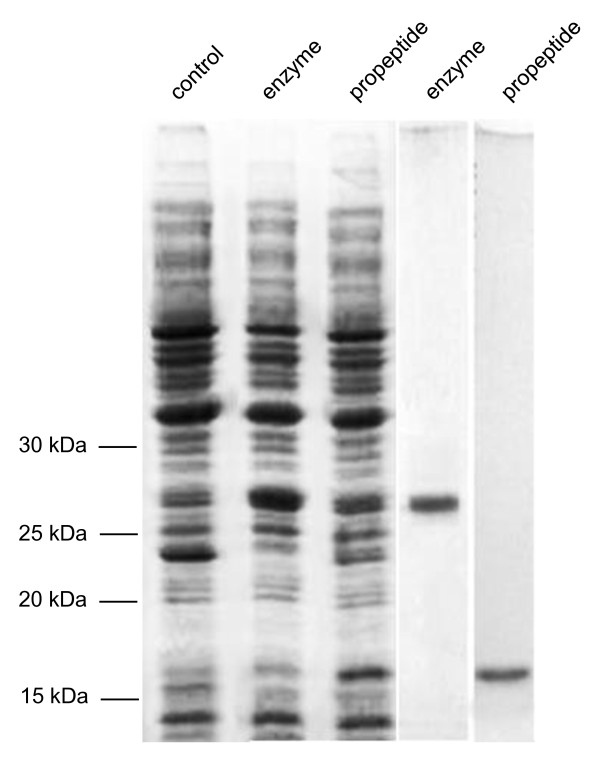
**Protein separation**. Left panel: Coomassie stained proteins from *E. coli *prior to induction (control) or after a 2 hour induction separated by SDS gel electrophoresis (enzyme: *E. coli *expressing the catalytic protein; propeptide: *E. coli *expressing the propeptide). Right panels: purified cuscutain (enzyme) or propeptide.

**Figure 4:**
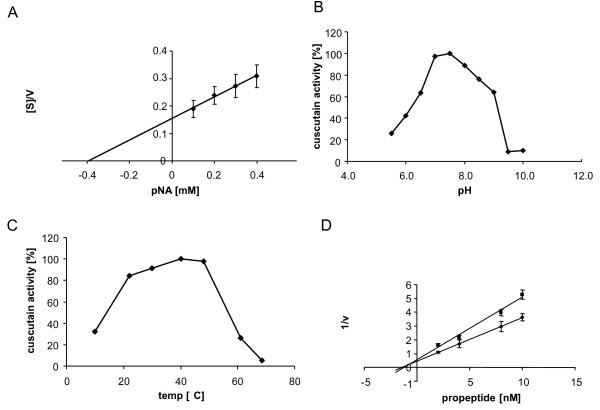
**Biochemical characterization of cuscutain**. **A **Hanes-Woolf plot of cuscutain activity for Km estimation. **B **relative cuscutain activity at different pH conditions. **C **relative cuscutain activity at different temperatures. **D **Dixon plot of cuscutain activity for estimation of inhibitor K_i_.

The coding sequence for the propeptide inhibitor region and that for the enzymatic activity were separately cloned into an *E. coli *expression vector. After induction, both proteins were expressed in *E. coli *and could be identified in crude extracts of soluble proteins by SDS gel electrophoresis and Coomassie staining. Nickel-column-chromatography was employed to purify the C-terminal 6xHis tagged proteins and provided a protein solution without significant impurities in the case of the propeptide as demonstrated by SDS gel electrophoresis and Coomassie staining (Figure 3). For the enzymatic protein portion a further purification step after gel-electrophoresis was applied as outlined. Eventually, also the enzymatic protein part was processed to purity as indicated by the staining of the corresponding gel (Figure 3). The purified enzymatic protein component was subjected to enzymatic characterization and the putative inhibitor component was tested for inhibitor efficiency. The results are summarized in Figure 4 and revealed for the *Cuscuta *cysteine proteinase a K_m _= 0,393 ± 0,02 mM or a turnover number of 3.5 aniline bonds/s (Figure 4A), an optimum at pH 7.0- 7.5 (Figure 4B) and a temperature optimum at 40°C (Figure 4C). These figures are in a comparable range to other cysteine proteinases (Table 1). In accordance to the naming of the papaya cysteine proteinase papain, the *Cuscuta *enzyme was denominated cuscutain. For the predicted inhibitory propeptide a K_i _of 0.7 ± 0.02 nM was determined on cuscutain activity (Fig 4D). The inhibitory effect on other cysteine proteinases like papain or cruzipain was found to be 20 times lower indicating a cuscutain-specific inhibitory function. The data support the sequence predictions concerning the biochemical function for both the enzyme and the inhibitor component.

**Table 1:** Biochemical characteristics of cysteine proteinases

	Cuscutain	Cruzipain	Papain
temperature Optimum [°C]	40	37	25
pH	7,5	8,0	6,5
K_m _[mM]	0,4	0,003	0,4

Biochemical characteristics of cuscutain, cruzipain [49] and papain [50,51].

### Biological function of cuscutain

**Figure 5:**
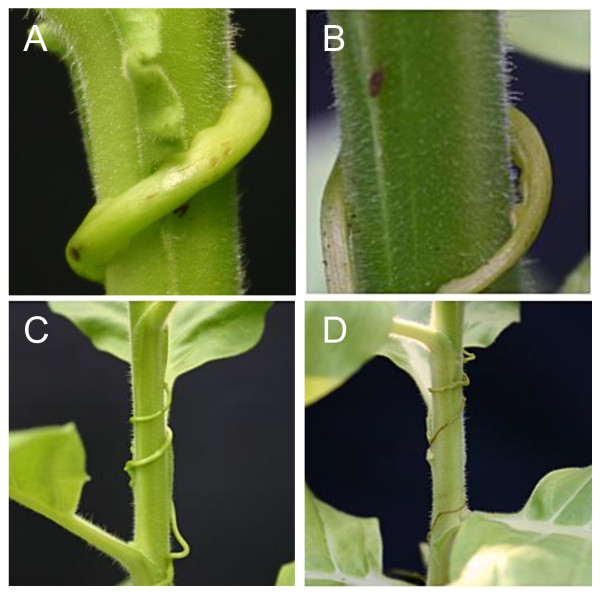
***Cuscuta reflexa *on tobacco**. The parasite successfully attached to untreated plants **(A, C)**, but not propeptide solution treated plants **(B, D)**.

Since cuscutain-mRNA was abundant in haustoria, a function of the encoded proteinase for the infection process was assumed. If cuscutain activity is essential for a successful infection, inhibition of the enzyme could be one way of reducing the effectiveness or preventing *Cuscuta *infestation. To test this assumption, 60 tobacco plants were prepared for infection by curling 20 cm *Cuscuta *shoot segments around the host shoot. One half of the assay was sprayed with 100 μg/ml buffered inhibitor propeptide solution 2 times a day for one week. As a mock control the other 30 plants were treated with buffer solution only. The propeptide solution had no visual effect on host tobacco plants e.g. with regard to development. On a daily basis, the plants were monitored for prehaustoria, haustoria and attached haustoria. Parasites on inhibitor propeptide solution treated *Cuscuta *- host plants appeared to be thinner and less vital as compared to controls (Figure 5A, B). For a quantitative assessment, the number of prehaustoria and haustoria attached or not attached to the host, were counted and related to *Cuscuta *shoot length. Untreated plants developed on average 9 (± 2.1) haustoria per 10 cm, 6 (± 1) of these produced a successful connection via hyphae. Inhibitor treated plants had 5.6 (± 1.6) haustoria per 10 cm with 1.5 (± 0.5) successful penetrations and connections to the host vascular tissue. On average treatment with the inhibitory propeptide solution reduced haustoria formation roughly by 40% and decreased successful parasite infestation from 65% down to 15%. In most cases (29 out of 30), *Cuscuta *on treated plants dried out after about two weeks without further spraying (Figure 5c, d), which suggests that a minimum number of successful connections to host xylem and phloem per parasite shoot length is required for vitality and propagation of *C. reflexa*. It also indicates that an active cuscutain cysteine proteinase is a component of a successful *Cuscuta *infestation.

## Discussion

A role of cuscutain during the infection process is suggested by the presented data. Accordingly, the sequence of related molecular events could be envisaged as follows: The cuscutain gene is activated concomitant to haustoria formation. The gene encodes a so called pre-pro-protein, with each of the protein subunits having a separate function. The prepeptide targets the cuscutain primary protein to the extracellular space. Here the unprocessed translation product is cleaved and deleted from the pre- and propeptide. Deletion of the inhibitor-propeptide converts cuscutain from an inactive form to an active enzyme with a cysteine proteinase function. Outside the parasite, the enzyme fulfills a role in the successful infection process, possibly by weakening host structures through protein degradation. Therefore, addition of water inhibitor solution by spraying most likely restricts this enzymatic activity outside the haustorial cells. It is yet unclear how a host-specific cuscutain activity is achieved, since the enzyme is most likely localized in the vicinity of host and *Cuscuta *tissue. If a concentration gradient of effective inhibitor components from parasite to host is created from primary cuscutain processing, the enzyme would show higher activity close to the host. Protective structures, e.g. the high degree of pectins on haustoria surfaces, could be another factor favoring the degradation of host cells in comparison to parasite tissue.

It is assumed that degrading enzymes, which are either parasite- or host-encoded support the penetration of parasitic hyphae [24,25]. In this regard the role of cuscutain resembles that of Orobanche encoded enzymes which were located in the cytoplasm and cell walls of intrusive cells and in the adjacent host apoplast during haustorium penetration [26-29].

## Conclusions

Papain-like cysteine proteases have been identified at the surface of various interaction surfaces between plants and pathogens like bacteria, fungi, oomycetes, nematodes insects or herbivores [30-40]. Some of these are a component of a defense mechanism while others are implicated in the parasitic pathogenic attack [41]. The identification of the cysteine proteinase cuscutain as a component that may be important for successful infestation of the parasitic plant *C. reflexa *could open the possibility for a new approach for development of parasitic plant blocking agents. During the parasite - host interactions both plant species act and react in order to invade, prevent, or tolerate invasion. Among others, these responses are visible as differential gene expression [42]. The identification of the corresponding proteins increases our knowledge about the molecular events of plant parasite infection. As demonstrated, the encoded proteins could also be significant for the host parasite interaction. There is a chance that a reduction of parasite-derived proteins weakens the parasite's infection efficiency and thereby strengthens host defense. However, prior to an application of a cuscutain propeptide solution in farming to protect crops some uncertainties must be ruled out. The inhibitor studies showed that its action spectrum is quite specific for cuscutain. It is unknown how similar cysteine proteases from other *Cuscuta *species or other parasitic plants are affected or if the inhibitor is effective on related proteases. It is possible that only one inhibitor is effective per species. On the other hand, the activity of cysteine proteinases could play a role in other parasitic plant interactions such as those with *Orobanche *or *Striga*. Although the latter parasitic weeds are root- and not shoot-parasites, the possibility of consistencies at the molecular level exists. Inhibition of cysteine proteases could thus be of wider importance for antagonizing parasitic plants from different genera.

## Methods

### Plant material and growth conditions

Tobacco plants were grown in standard potting soil under 16 h/8 h day/night light conditions (800 μmol photons m^2 ^s^-1^) at 25 °C. *Cuscuta reflexa *was grown on *Coleus blumei *host plants under the same greenhouse conditions as the tobacco plants. *Coleus blumei *was chosen as host plant for *Cuscuta *cultivation because it tolerated this parasitic infection. *C. reflexa *was propagated vegetatively throughout. For infection of tobacco plants, *C. reflexa *shoot tips of about 20 cm length were wrapped around a wooden stick. After 24 h, *C. reflexa *shoots were transferred to 4-5-week-old tobacco plants and curled around the stem; this time point was set as the starting point of the infection process.

### Northern blot analysis

RNA isolation from *Cuscuta *shoots was performed using the RNAeasy plant mini kit (Qiagen) following the manufacturer's instructions. The gel was loaded with 5 μg RNA per lane and blotted onto a nylon membrane (Applichem Inc., Darmstadt, Germany). The blot was hybridized with a cDNA probe comprising 270 bp of the open reading frame encoding the Cuscutain-enzyme active site. It was synthesized by PCR (forward primer: 5'-GGCGCGCCCCATACATTTGCTCCAAGCGG-3'; reverse primer: 5'-ATTTAAATGTGCTAACAGCTGCCACAGTTG-3'). The PCR reaction was carried out using DIGlabelled dUTPs. Membrane blots were pre-hybridized for 2 h in DigeasyHyb (Roche Diagnostics GmbH, Mannheim, Germany); subsequently, the denatured probe was added for overnight hybridization at 42 °C. Washing and detection was performed following the manufacturer's protocol. Detection was carried out following the suppliers protocol (Pharmacia Biotech, Munich, Germany) using an alkaline phosphatase-conjugated antibody and CDPstar as substrate for chemiluminescence reaction. The chemiluminescence was visualized and quantified using a chemiluminescence detector equipped with a digital camera and quantification software (BioRad).

### Comparative macroarray

Tissue samples of *Cuscuta reflexa *including prehaustoria and haustoria were collected 3 days after the infection of 5 weeks old tobacco plants. Total RNA was isolated using RNAeasy plant mini kit (Qiagen, Germany) following the manufacturer's instructions. 2 μg total RNA was employed for first strand cDNA synthesis (Ready-To-Go^™ ^ You-Prime First-Strand Beads; GE Healthcare) using 3'CDS-primer and SMARTIIA-oligo-primer from SMART^™ ^cDNA synthesis system (Clontech). cDNA was PCR amplified applying the above mentioned primers and the product was cloned via TA cloning (TOPO TA Cloning^®^, Invitrogen). Colonies of transformed bacteria were selected on Ampicillin (50 μg/ml) containing agar plates using blue/white selection (LB-agar plates: 1% NaCl, 1% Trypton, 0.5% yeast extract, 1.5% agar, 60 μg/ml Isopropyl-β-D-thiogalactopyranosid, 40 μg/ml X-Gal). White colonies were transferred into a 96 well plate, each well filled with 200 μl LB (1% NaCl, 1% Trypton, 0.5% yeast extract), grown overnight and stored after addition of one drop of glycerol (90%) at -80°C. An aliquot of the E.coli stock (10 μl) was subjected to PCR amplification of the plasmids cDNA insertion using vector specific primers and following standard procedures to a product end concentration of 70- 100 ng/μl in a 96 well plate. Using a Microcaster^™ ^device (Microcaster^™^, Schleicher & Schüll) 768 amplified cDNAs were stamped to a Castslide^™ ^(Schleicher& Schüll) having an area of about 2 cm². After the punctual application of cDNAs the slides were treated with denaturing solution (0.4 M NaOH, 3 × SSC, 10 mM EDTA) for 5 minutes and then with a neutralizing buffer (0.5M Tris-HCL pH 7.0, 1.5 M NaCl). For the differential hybridization a single stranded cDNA probe from total RNA of *Cuscuta reflexa *shoot material without prehaustoria and haustoria or with prehaustoria and haustoria was labelled using Label Star Array Kit (Quiagen) and ³³P.dCTP. After incubation for 1 h at 42 °C with PreHyb/Wash Buffer (CAST^™ ^MicroHybridization Kit, Schleicher & Schüll) the slides were incubated in a volume of 1 ml and 1 million cpm labelled cDNA over night at 42 °C. The slides were washed 3 times for 30 minutes at room temperature with PreHyb/Wash Buffer and the hybridization signals detected and quantified with a Phoshorimager (BAS-1800 Scanner, BasReader, Fuji). 7000 cDNAs were screened and 16 corresponding genes that were identified to be clearly upregulated in the haustoria containing fraction were identified.

#### Sequence comparison and predictions

Sequence based comparisons with the Cuscutain cDNA were performed by web-based tools at NCBI (http://www.ncbi.nlm.nih.gov). Cleavage site prediction of the deduced Cuscutain pre-pro-protein was determined by application of Protein Machine software available at Expasy (http://us.expasy.org/tools/).

### Expression and isolation of the propeptide and cuscutain

The plasmid for the expression of the propeptide inhibitor fragment (amino acid residues 32-134) and the cuscutain enzymatic region (amino acid residues 135-367) were constructed using the GATEWAY^™^-system (Invitrogen) according to the manufacturer's protocol [43]. Entry vector pDonr201 and destination vector pETDest42 (Invitrogen) were used for cloning the respective Cuscutain cDNA or cDNA fragment in frame with a 6 x His tag at the protein's C-terminus. The propeptide containing polypeptide and that with cuscutain were expressed in *E. coli *(BL21Lys). 100 ml of *E. coli *suspension was grown in an appropriate Erlenmeyer flask in LB [44] at 37 °C. At an O.D. of 0.8, the 1 mM IPTG was added for 4 hours and the suspension centrifuged for 10 min at 13.000 rpm. The supernatant was discarded and the cells resuspended in 4 ml of resuspension buffer (50 M Na-phosphate, pH 7.5, 4 M urea, 300 mM NaCl). The HIS-tagged propeptide was allowed to bind to pre-equilibrated BD TALON^™ ^metal affinity resin (Clontech) overnight at 4 °C. Subsequently, the resin was washed 3 times with resuspension buffer. Bound protein was eluted using 1 ml of elution buffer (50 M Na-phosphate, pH 7.5, 4 M urea, 300 mM NaCl and 150 mM Imidazole). Prior to further analysis, the obtained solution was dialysed against 50 mM Na-phosphate, pH 7.5. *E. coli *expressing cuscutain were resuspended in 1.5 ml electrophoresis buffer (0.25 M Tris-HCl, pH6.8, Glycerol 50%, SDS 0.2 g), subjected to sonification and separated by SDS-gel electrophoresis using standard methods [45]. By comparison to a parallel Coomassie stained gel, the lanes containing cuscutain were identified and cut out. The HIS tagged cuscutain enzymatic region was eluted using the Elutrap system (Schleicher & Schüll) following to the manufactures' protocol. Elution was performed overnight at 80 V. SDS was removed from the sample using the method of Henderson et al.[46]. To ensure cuscutain enzymatic a buffer containing 40 mM Tris/borate, pH 8.5, 50% glycerol, 3 mM glutathione was added drop wise to a sample dilution of 200 : 1 and incubated overnight at 4 °C according a protocol provided by Tobbell et al [47]. For protein concentration, the solution was electro eluted again at 200 V. Purified proteins were stored at -20°C.

### Kinetic Measurements

Cuscutain activity was determined using the colorimetric papain substrate Cbz-Phe-Arg-pNA. In brief 12.08 μM cuscutain and 0.4 mM dipeptide in 500 μl 0.1 M Na-citrate, pH 7.5, containing 20% ethanol, were incubated at 37°C for 10 min. After addition of 500 μl 5 mM PMSF in DMSO, absorbance of *para*-nitroaniline was measured at a wavelength of 405 nm. Cbz-Phe-Arg-pNA concentrations of 0.2 mM and 0.4 mM were used for 1/v versus [I] plots [48]. All measurements for cuscutain were performed at 30 °C, and assay conditions were 50 mM Tris-buffer, pH 7.5 containing 300 mM NaCl. The presented data rely on three independent experiments throughout.

## Author's contributions

MB: Isolated and characterized cuscutain. Performed biological significance tests. MA: Constructed cDNA-library and screening. LF: Significantly contributed to enzymatic characterization of cuscutain. RK: Contributed to conception and design, analysis and interpretation of data. Was involved in drafting the manuscript and revising it critically for important intellectual content. All authors read and approved the final manuscript
